# Evaluation of lifestyle interventions to treat elevated cardiometabolic risk in primary care (E-LITE): a randomized controlled trial

**DOI:** 10.1186/1471-2296-10-71

**Published:** 2009-11-12

**Authors:** Jun Ma, Abby C King, Sandra R Wilson, Lan Xiao, Randall S Stafford

**Affiliations:** 1 Palo Alto Medical Foundation Research Institute, Palo Alto, CA, USA; 2 Stanford Prevention Research Center, Stanford University School of Medicine, Stanford, CA, USA; 3 Department of Health Services Research, Palo Alto Medical Foundation Research Institute, 795 El Camino Real (Ames Bldg.), Palo Alto, CA 94301, USA

## Abstract

**Background:**

Efficacy research has shown that intensive individual lifestyle intervention lowers the risk for developing type 2 diabetes mellitus and the metabolic syndrome. Translational research is needed to test real-world models of lifestyle interventions in primary care settings.

**Design:**

E-LITE is a three-arm randomized controlled clinical trial aimed at testing the feasibility and potential effectiveness of two lifestyle interventions: information technology-assisted self-management, either alone or in combination with care management by a dietitian and exercise counselor, in comparison to usual care. Overweight or obese adults with pre-diabetes and/or metabolic syndrome (n = 240) recruited from a community-based primary care clinic are randomly assigned to one of three treatment conditions. Treatment will last 15 months and involves a three-month intensive treatment phase followed by a 12-month maintenance phase. Follow-up assessment occurs at three, six, and 15 months. The primary outcome is change in body mass index. The target sample size will provide 80% power for detecting a net difference of half a standard deviation in body mass index at 15 months between either of the self-management or care management interventions and usual care at a two-sided α level of 0.05, assuming up to a 20% rate of loss to 15-month follow-up.

Secondary outcomes include glycemic control, additional cardiovascular risk factors, and health-related quality of life. Potential mediators (e.g., treatment adherence, caloric intake, physical activity level) and moderators (e.g., age, gender, race/ethnicity, baseline mental status) of the intervention's effect on weight change also will be examined.

**Discussion:**

This study will provide objective evidence on the extent of reductions in body mass index and related cardiometabolic risk factors from two lifestyle intervention programs of varying intensity that could be implemented as part of routine health care.

**Trial registration:**

NCT00842426

## Background

Diabetes mellitus affects nearly 24 million Americans (8% of the U.S. population), and type 2 diabetes accounts for about 90% to 95% of all diagnosed cases of diabetes [1,2]. The prevalence of diagnosed type 2 diabetes increased six-fold in the latter half of the past century [1,2]. This dramatic increase is largely attributed to the epidemics of obesity and physical inactivity [3]. About two-thirds of U.S. adults are overweight or obese [4], and 57 million people are estimated to have pre-diabetes -- a condition characterized by blood glucose levels that are higher than normal but not yet high enough to be diagnosed as diabetes. Overweight or obese individuals with pre-diabetes are at high risk for progression to diabetes. A vast majority of these individuals also have an increased risk of cardiovascular disease (CVD) because of concomitant risk factors, such as abdominal obesity, atherogenic dyslipidemia, and elevated blood pressure. The constellation of these common CVD risk factors is known as the metabolic syndrome [5].

The Diabetes Prevention Program (DPP) has demonstrated that an intensive lifestyle intervention focused on lifestyle changes and weight reduction can successfully prevent or delay the onset of type 2 diabetes and the metabolic syndrome in overweight or obese adults with pre-diabetes [6]. The DPP intervention required frequent, face-to-face, individual counseling [7]. Despite evidence of potential cost-effectiveness of the DPP intervention [8,9], the substantial resources required for its implementation are a significant barrier to widespread dissemination. A wide range of research has been underway related to translation of the DPP into real world settings, such as studies that assess cost-effective and generalizable methods of delivering evidence-based lifestyle modifications in small groups and over the Internet, as well as methods to sustain behavior change and weight loss [10-12].

Recently, the 16 "core curriculum" individual counseling sessions of the DPP intervention have been adapted for delivery in a 12 session group-based program called Group Lifestyle Balance (GLB)™ [13], and preliminary evaluations of the program have suggested potential effectiveness [14,15]. Past studies have shown that group interventions are similar in efficacy to individual interventions for weight loss [16,17] and may markedly improve intervention cost-effectiveness [8,9]. The Internet is another alternative mode of delivery of lifestyle interventions. Several randomized controlled trials have demonstrated the efficacy of Internet-based lifestyle interventions in overweight or obese individuals [18-20], including those at risk for type 2 diabetes [21].

To date, few studies have rigorously tested the feasibility and effectiveness of group- and Internet-based lifestyle interventions for weight management and risk reduction in primary care settings. Weight loss in overweight or obese patients with associated risk factors is of high priority for clinical care settings given the growing prevalence of obesity-related complications, and preventative health care is a major primary care goal. However, obesity and risk factor management has thus far been largely a failure in primary care settings throughout the United States [22,23]. Research is needed to translate efficacious lifestyle interventions into primary care practice, and in the process, evaluate their cost-effectiveness, generalizability, and sustainability.

### Aims

The present study aims to evaluate the feasibility and potential effectiveness of two lifestyle interventions, information technology-assisted self-management alone (the SM intervention) or SM combined with care management by a dietitian and exercise counselor (the CM intervention), in comparison to usual care (UC). The primary hypothesis for each intervention (SM and CM) is that intervention participants will achieve greater reductions in body mass index (BMI) than UC participants at 15 months. Secondary research goals include comparing the effects of each intervention to UC on changes in glycemic control (fasting blood glucose and hemoglobin A1c), additional cardiovascular risk factors (serum lipids, blood pressure, waist circumference, and C-reactive protein [CRP]), health-related quality of life and psychosocial well-being. Differences between CM and SM intervention effects on weight loss and secondary outcomes also will be examined. Potential mediators (e.g., treatment adherence, caloric intake, physical activity level) and moderators (e.g., age, gender, race/ethnicity, baseline mental status) of the intervention effects on weight change and secondary outcomes will be explored. Additionally, process measures will be evaluated to assess the extent of participation of patients in each intervention and the potential reach, adoption, implementation and maintenance of the interventions in primary care practice.

## Methods/Design

### Study Design

E-LITE is a randomized controlled clinical trial in which overweight or obese adults with an increased risk for CVD and diabetes are equally randomized to one of three arms: UC (control), SM, or CM. All procedures and materials were approved by the Palo Alto Medical Foundation Research Institute's Institutional Review Board.

### Eligibility Criteria

Participants are recruited from a primary care health center of the Palo Alto Medical Foundation (PAMF), a community-based multi-specialty group practice in California. Primary care patients ages 18 years and older who are overweight or obese and have pre-diabetes and/or metabolic syndrome are eligible to participate. Exclusion criteria are designed to: 1) avoid potential contamination and minimize confounding; 2) minimize safety concerns; and 3) prevent poor adherence and loss to follow-up. Table 1 enumerates the inclusion and exclusion criteria.

**Table 1:** Participant inclusion and exclusion criteria

**Inclusion criteria**
1. Ethnicity: All ethnic groups;
2. Gender: Men and Women;
3. Age (as of date of enrollment):
a). Lower age limit: 18 years;
b). Upper age limit: NONE (*only exclude for cause, e.g. diseases, functional limitations detailed below*);
4. Body mass index ≥ 25.0 kg/m^2^;
5.Having pre-diabetes and/or metabolic syndrome based on the following criteria:
a). Pre-diabetes: fasting plasma glucose between 100 and 125 mg/dL;
b). Metabolic syndrome: Three or more of the following:
--Waist circumference ≥ 40 inches in men; ≥ 35 inches in women (if in Asian American ≥ 35 inches in men; ≥ 31 inches in women);
--Triglycerides ≥ 150 mg/dL;
--High-density lipoprotein cholesterol (HDL-C) < 40 mg/dL in men; < 50 mg/dL in women;
--Systolic blood pressure ≥ 130 mm Hg or diastolic blood pressure ≥ 85 mm Hg;
--Fasting plasma glucose between 100 and 125 mg/dL.
6. Having a primary care physician (PCP);
7. Able and willing to enroll and provide written, informed consent, i.e., to: 1) meet the time and data collection requirements of the study; 2) be randomized to one of the three intervention arms; 3) adhere to the recommendations of the study intervention as assigned; 4) participate in follow-up for 12 months; and 5) allow extraction of relevant information from their medical records.
**Exclusion criteria**
1. Inability to speak, read or understand English;
2. No regular access to a computer with Internet and email capabilities;
3. Triglycerides ≥ 400 mg/dL;
4. Systolic blood pressure ≥ 160 mm Hg or diastolic blood pressure ≥ 100 mm Hg;
5. Initiation or change of drug therapy for elevated blood pressure or abnormal lipid levels within the past 3 months;
6. Initiation or change of antidepressant medication within the past 3 months;
7. Having a medical or physical condition that make moderate intensity physical activity (like a brisk walk) difficult or unsafe;
8. Use of weight-loss medications in the past 3 months;
9. Regular use (> 5 days/month) of medications that affect appetite or weight (e.g., oral corticosteroids, insulin, oral hypoglycemics etc.);
10. Currently enrolled in a lifestyle intervention program at PAMF or elsewhere;
11. Planning to undergo bariatric surgery during the study period;
12. Diagnosis of Type 1 or Type 2 diabetes mellitus;
13. Significant medical co-morbidities, including uncontrolled metabolic disorders (e.g., thyroid, renal, liver), heart disease, stroke, and ongoing substance abuse;
14. Renal insufficiency (i.e. glomerular filtration rate < 60 mL/min/1.73 m^2^)
15. Diagnosis of psychiatric disorders that would limit adequate informed consent or ability to comply with study protocol;
16. Diagnosis of cancer (other than non-melanoma skin cancer) that was active or treated with radiation or chemotherapy within the past 2 years;
17. Diagnosis of a terminal illness and/or in hospice care;
18. Pregnant, lactating or planning to become pregnant during the study period;
19. Already enrolled or planning to enroll in a research study that would limit full participation in this study or confound the observation and interpretation of the study's findings;
20. Family/household member of another study participant or of a study staff member;
21. No longer a PAMF patient or planning to transfer care outside of PAMF during the study period;
22. Planning to move out of the area during the study period;
23. PCP determination that the study is medically inappropriate or unsafe for the patient;
24. Investigator discretion for clinical safety or protocol adherence reasons.

### Recruitment and Screening Process

Target sample size for E-LITE is 240, recruited in three sequential cohorts. The screening process for each cohort begins with identification using relevant data available in the patient's electronic health record of age-appropriate, potentially eligible patients whose primary care provider (PCP) regards the interventions under study to be medically appropriate and safe for that patient. Patients whose participation is approved by their PCP are asked to complete a brief questionnaire regarding major exclusions, either online or by phone. Those who pass the initial screen are scheduled for a formal eligibility determination (baseline) visit (BV1), and, within two weeks prior to the visit, they are contacted and given instructions for completion of an online baseline questionnaire.

The BV1 occurs no more than four months after the initial screen and no more than four weeks prior to randomization. It includes completion of additional eligibility questionnaires, measurement of height, weight, waist circumference, and resting blood pressure, and a fasting blood draw for tests of plasma glucose and lipids at the onsite Clinical Laboratory. Time permitting, a seven-day physical activity recall [24] is completed; otherwise, it will be completed at the baseline assessment/randomization visit (BV2). Participants are given instructions and a three-day food record form to be completed and returned at BV2. Participants who have possible angina or peripheral vascular disease based on the Rose Questionnaire [25] are referred to their PCP for further evaluation and may only continue with explicit authorization of their physician.

Participants who are eligible to continue on the basis of BV1 are scheduled for BV2, which occurs at least seven days after BV1 and no more than three weeks prior to the start of the intervention. The BV2 includes measurement of weight, waist circumference, and resting blood pressure, a review of the three-day food record for completeness, complete review of medication and supplement use, a non-fasting blood draw for tests of A1c and CRP, and if necessary, completion of the physical activity recall. Following the measurements, participants are randomized.

All participants are properly and adequately informed and give appropriate consent at each step in the screening process of the trial and prior to participating in the post-randomization phase.

### Randomization and Blinding

Participants are randomized on a 1:1:1 basis to one of three arms: UC, SM, or CM. Pocock's "minimization" procedure [26] is used to assure better than chance group balance with respect to participant age, gender, race, BMI, fasting blood glucose, waist circumference, and use of PAMFOnline, which is PAMF's online patient portal to access his or her own health record (user vs. non-user). For each participant about to be randomized, a computerized randomization algorithm automatically calculates an imbalance score for each of the balancing factors, as the excess or deficit of previously randomized participants in each arm matching the current patient on that factor. These scores are summed over factors to form a total imbalance score, S, for each treatment arm. The randomization probability of assigning the patient to the treatment associated with the smallest S is set to 2/3, and the other two treatments are each assigned a probability of 1/6 based on Efron's biased coin method [27].

A designated research staff member who is not involved in follow-up data collection or data analysis assigns each study arm a non-revealing label, e.g., A, B, or C, and performs actual randomization of the participants. These labels are used in all study documents and other materials to ensure blinding of the investigators and staff responsible for follow-up data collection and analysis to participant treatment assignments throughout the trial.

### Baseline and Follow-up Measures and Data Collection

Table 2 shows a complete list of study measures and the data collection schedule. The time commitment of participants is approximately 2 hours 45 minutes at baseline (for completing the initial screen, baseline questionnaire, BV1 and BV2), 45 minutes for the 3-month assessment, and 1 hour 30 minutes each for the 6-month and 15-month assessments.

**Table 2:** List of measures and data collection schedule

		Follow-up Month		
	Baseline	3	6	15
**Clinical Measures**				
Height	X			
Weight	X	X	X	X
Waist circumference	X	X	X	X
Blood pressure	X	X	X	X
Fasting blood: Total cholesterol, LDL-C, HDL-C, triglycerides, glucose, hemoglobin A1c	X		X	X
C-reactive protein (CRP)	X			X

**Questionnaires**				
Demographics	X			
Family medical history	X			
Three-day food record	X	X	X	X
Eating Habits Confidence Survey	X	X	X	X
Social Support and Eating Habits Survey	X	X	X	X
Stanford Seven-day Physical Activity Recall	X	X	X	X
Exercise Confidence Survey	X	X	X	X
Social Support and Exercise Survey	X	X	X	X
Smoking and alcohol consumption	X			X
12-item Short Form Health Survey (SF-12)	X	X	X	X
Obesity Related Problems Scale	X	X	X	X
Nine-item Patient Health Questionnaire (PHQ-9)	X	X	X	X
Depression Anxiety Stress Scale	X	X	X	X
Symptoms	X	X	X	X
Adverse events		X	X	X
Medication use	X	X	X	X
Care at non-PAMF health care facilities	X	X	X	X
Unified Theory of Acceptance and Use of Technology^1^		X	X	X
**Data extracted from PAMF electronic databases**				
Current medical problems	X			X
Medications prescribed	X			X
Health care utilization	X			X
PCP characteristics	X			

^1^Questionnaire administered to participants in the SM and CM interventions only

Abbreviations: HDL-C, high-density lipoprotein cholesterol; LDL-C, low-density lipoprotein cholesterol; PCP, primary care provider.

#### Primary and Secondary Outcomes

The primary outcome measure is the BMI. Height and weight, in light indoor clothes without shoes, are measured using a wall-mounted stadiometer and a balance beam scale, respectively. All scales are calibrated quarterly by trained study personnel using standard weights. BMI is calculated as the Quetelet index (kg/m^2^).

Secondary outcomes include glycemic control and component risk factors of the metabolic syndrome. Plasma glucose, total cholesterol, triglycerides, and high-density lipoprotein cholesterol (HDL-C) are measured after an overnight fast, and low-density lipoprotein cholesterol (LDL-C) is calculated by the Friedewald equation [28]. Hemoglobin A1c and CRP are also measured. Waist circumference is measured using a tape, according to a standardized protocol, in a horizontal plane around the abdomen at the level of the right iliac crest[29] Blood pressure is measured in the seated position after at least a five-minute rest. At the BV1, blood pressure is measured in both arms and the arm with the higher pressure is used for all subsequent blood pressure measurements. At each assessment visit, three blood pressure measurements are obtained with one-minute between measurements, using equipment and procedures that meet the American Heart Association's recommendations for blood pressure measurement in humans [30].

Additional secondary outcomes include generic and obesity-specific health-related quality of life and mental health. The 12-item Short Form Health Survey (SF-12) [31], a widely used quality of life instrument, is administered to evaluate changes in non-disease specific physical and mental health status. The Obesity-Related Problem Scale specifically measures the impact of obesity on psychosocial functioning. The eight-item scale has high internal reliability and sound test--retest reliability, correlates strongly with a wide range of theoretically related constructs, and is responsive to weight loss intervention [32]. The nine-item Patient Health Questionnaire (PHQ-9) refers to the previous two-week interval and consists of nine items of depression symptoms and one follow-up question on functional impairment [33]. The PHQ-9 is both a measure of depressive symptomatology and a tentative diagnostic instrument for the *Diagnostic and Statistical Manual of Mental Disorders*, 4^th ^edition (DSM-IV) depressive disorders [34]. The 21-item Depression Anxiety Stress Scale has high internal consistency and correlates strongly with other validated self-report measures of depression, anxiety, and stress [35].

Furthermore, changes in diagnoses, medication use and health care utilization will be assessed. With participant authorization, data are extracted from their EHRs at baseline and again at 15 months on current medical problems, current prescription medications, and health care contacts during the study (including outpatient visits, ER visits, hospitalizations, and phone/email consultations). Pharmacy dispensing data are currently available for patients with capitated insurance only (making up about 30% of the PAMF patient population). While the vast majority of health care of PAMF primary care patients, including specialist care, is received from PAMF, a significant proportion of medications prescribed are not obtained from the PAMF pharmacy. Also, PAMF patients may seek care outside the system. Therefore, additional data are obtained on medication and supplement use by recording information from the container and on health care outside PAMF by participant self-report.

#### Potential Mediators

Data are also obtained on a battery of behavioral and psychosocial measures using existing self-report instruments to assess potential mediators of the interventions.

The two main foci of the SM and CM interventions are dietary changes to achieve weight loss and increased physical activity. Adherence to the interventions is assessed by three-day food records and the Stanford 7-Day Physical Activity Recall. Multiple-day food records are considered a gold standard for collection of individual dietary data [36]. The Stanford 7-Day Physical Activity Recall has been tested for validity and reliability [24] and is sensitive to change in physical activity [37,38]. In addition, adherence to the overall intervention "process" is assessed by documenting attendance at group sessions, completion of self-monitoring records, and frequency of use of the online self-management support systems included in the SM and CM interventions. Self-monitoring data obtained during the intervention program also are used for adherence monitoring and for individual feedback.

Based on the social cognitive theoretical underpinnings of the intervention (see below), two main hypothesized mediators of the intervention are selected to be measured: self-efficacy and social support. The Eating Habits Confidence Survey and the Exercise Confidence Survey [39], and the Social Support and Eating Habits Survey and the Social Support and Exercise Survey [40], have been shown to be reliable and have high internal consistency and are associated with other measures of social support and self-efficacy related to physical activity and dietary behaviors.

The questionnaire created by Venkatesh et al. [41] based on the Unified Theory of Acceptance and Use of Technology, a technology acceptance model, is adapted to assess user acceptance and use of the online self-management support systems among SM and CM intervention participants. The questionnaire includes the following scales: Performance Expectancy, Effort Expectancy, Social Influence, Facilitating Conditions, Attitude toward use, Self-Efficacy, Anxiety and Behavioral Intention.

#### Additional Process Measures

Data on the proportion and representativeness of patients willing to participate, as well as reasons for declining to participate, are used to assess the potential reach of the E-LITE interventions. Demographic and professional characteristics of PCPs (e.g., age, sex, years in practice, specialty and board certification, PAMF department, panel size and composition, %FTE) are obtained from PAMF administrative records. Data on the proportion and representativeness of physicians willing to approve their potentially eligible patients for participation are used to assess the likelihood of adoption of the E-LITE interventions.

### Interventions

#### Theoretical Basis

The theoretical underpinning of the E-LITE interventions is derived from Social Cognitive Theory [42] and the Transtheoretical Model of Behavior Change [43]. The former emphasizes the reciprocal determinism between individual, environment, and behavior, whereas the latter recognizes that behavior change is a dynamic process that moves, at variable speed, through varying stages of readiness to change. Behavior change is more likely with increased behavior capability, and behavior capability is strengthened through goal setting, skill building and self-monitoring. Also important are confidence in one's ability to perform a given behavior (self-efficacy) and expectation of favorable outcomes of the behavior (outcome expectations). Behavioral strategies may vary by stage of change (i.e., experiential processes during initial phases of behavioral adoption and behavioral processes occurring during action and maintenance phases) [43]. In addition, the E-LITE interventions draw upon key research evidence for the importance of self-management in chronic disease management [44-46]. Successful self-management intervention is driven by patient-defined problems and fosters the mastery of skills in problem solving, action planning, decision making, and support building through an iterative process [46,47].

This theoretical basis distinguishes the E-LITE interventions from the delivery of lifestyle modifications in the usual primary care setting. Current practice routinely involves physician advice, occasionally with referral to a dietitian who takes a diet history and who then provides standard educational materials and advice during a small number of individual or group sessions, typically with little or no follow-up. This standard clinical approach lacks the theoretical basis and behavioral self-management focus described above.

#### Evidence-based Intervention Goals

A goal-based approach is used in both SM and CM interventions, in which participants are given the same general goals: 7% weight loss and 150 minutes of moderate physical activity per week. These goals are consistent with those of the DPP [7]. Participants who wish to lose more than 7% of their starting weight may be encouraged to do so only insofar as the participant maintains a normal BMI at or above 21 and the rate of weight loss does not exceed three pounds per week. The physical activity goal is consistent with the new Physical Activity Guidelines for Americans [48], and is deemed safe and attainable for most adults. Participants, especially those who are habitually sedentary, are advised to gradually and steadily increase their activity level and reach the goal within five weeks of beginning activity, or as soon as possible thereafter. Regular physical activity recommendations for activities of moderate intensity other than brisk walking, as well as strength and flexibility physical activities, are to be tailored to participant situation [49,50]. After attaining the minimum goal of 150 minutes a week, participants who wish to be more active are encouraged to do so, as tolerated. If participants reach the 150-minute goal but are not achieving the weight goal, they are encouraged to further gradually increase their physical activity to 60 minutes/day of moderate physical activity [49,51,52]. Participants in both SM and CM interventions are strongly encouraged to track their weight and physical activity using the American Heart Association's online self-management portal at http://www.heart360.com.

Recommendations are also given for total fat reduction (to 25% of calories from fat) and calorie balance and restriction (with a goal of a 500- to 1000-calorie reduction diet). As in the DPP [7], the calorie and fat goals are given as a means to achieve and maintain the weight goal, rather than as a goal in and of itself. Therefore, if a participant consumes more than the assigned calorie or fat goal, but is achieving the weight goal, there is no need to focus on further reductions. Portion control, choices of low-energy and nutrient-dense meals and snacks, healthy food preparation techniques, and careful selection of restaurants, including fast food, and the items offered are promoted as strategies to gradually achieve the calorie and fat goals. Participants also are recommended to (1) lower saturated fat intake to < 10% of caloric intake, (2) lower cholesterol intake to < 300 mg/day, (3) consume a high plant-based diet that includes a variety of fruits and vegetables, whole grains, and low-fat dairy products, and (4) reduce intake of high glycemic index carbohydrates.

#### Intervention Format, Structure, and Content

Both SM and CM interventions last 15 months and involve Intensive Treatment and Maintenance phases.

#### Intensive Treatment Phase

The Intensive Treatment of both interventions lasts three months and is based on the GLB program [13]. There are 12 weekly sessions and each lasts 90-120 minutes. Each session follows a similar structure and includes five curriculum components: 1) measurement and recording of weight, 2) review of self-monitoring records and progress, 3) identification of personal barriers to weight loss and activity and potential solutions, 4) presentation of a new content area, and 5) goal setting and action plans for next week.

SM condition. After attending a single-session group orientation, participants in the SM intervention are given the GLB program on a DVD to follow at home, with access to a study dietitian via secure online messaging for advice and support. Secure messaging is integrated with the electronic health record and allows patients to communicate confidentially with their provider online. The DVDs are provided by the Diabetes Prevention Support Center at the University of Pittsburgh Diabetes Institute. Participants are encouraged to message the dietitian comments and questions as they are completing the DVD program. The dietitian will answer secure messages from participants within 1-2 work days and provide general support and encouragement.

CM condition. Participants in the CM intervention receive the GLB program in 12 weekly group sessions led by a study dietitian and exercise physiologist. Both interventionists have training and experience in weight management counseling, and the dietitian has completed the GLB 2-day training workshop offered by the University of Pittsburgh's Diabetes Prevention Support Center. The sessions are conducted in a highly interactive fashion. The GLB Manual of Operations is closely followed, except for the addition of a food tasting activity as part of the check-in process and a 30-minute physical activity demonstration at the end of each session. The food tasting activities are aimed at increasing participant experience with healthy food choices, encouraging them to try new foods that they might not normally try on their own, and stimulating a social, fun and engaging environment. The physical activity demonstrations are aimed at increasing participant awareness of physical activity intensity, experiencing various moderate-intensity activities that are safe and that they can easily incorporate into their daily lives, and increasing participant confidence. E-LITE participants in both CM and SM arms receive a pedometer in the initial session (the group orientation for the SM arm), as opposed to in session 10 as in the original GLB program [13].

#### Maintenance Phase

Both SM and CM interventions use secure online messaging as the primary mode of contact between participants and the dietitian during the maintenance phase. The contact time is likely to vary depending on individual needs in either intervention arm. The exercise physiologist is available to counsel the dietitian on issues related to physical activity but she will have no direct communication with participants in this phase. Participants are instructed to continue monitoring their weight, dietary intake, and physical activity at least twice each week.

SM condition. SM participants receive an email reminder every two weeks to continue self-monitoring. As in the Intensive Treatment phase, they are encouraged to submit comments and questions to the dietitian via secure messaging. The dietitian will respond to participant inquiries but will not initiate contact.

CM condition. In contrast, the dietitian provides active follow-up and individualized counseling to participants in the CM group via secure messaging on at least a monthly basis. Based on participant progress, the dietitian provides tailored feedback to reinforce progress, recommend problem solving and relapse prevention strategies, and encourage maintenance efforts. As the participant is successful or not in attaining his/her current goals, changes to the individual action plan are recommended, e.g., modifying goals, adding to them, or replacing them with new goals. The objective of any changes to the action plan is to keep the goals realistic yet challenging and to accommodate preferences and periodic fluctuations in motivation and schedule. Participants are encouraged to practice subject-initiated relapse prevention strategies, with feedback and support from the dietitian. Participants who fail to complete their self-monitoring diaries as scheduled are queried about their progress relative to their weight loss and behavioral change goals and any barriers they have encountered in attaining those goals. They are strongly encouraged to monitor and continue with the intervention. At the discretion of the dietitian, participants may be phoned or seen to address outstanding issues related to poor understanding of and/or adherence to the intervention protocol.

### Participant Safety

PCP approval is required before potentially eligible patients are contacted by the study. Participants are carefully screened and individuals for whom the interventions would be medically inappropriate or unsafe are excluded. During screening, women who are pregnant, lactating, or planning to become pregnant during the study period are excluded. If a participant becomes pregnant during the study, she is excluded immediately from further participation in all study activities, and her PCP is immediately notified. Participants who develop any other exclusionary condition (e.g., diabetes) following randomization may continue with the interventions and follow-up assessments only after explicit approval of their PCP. Surveillance for adverse events and relevant clinical events occurs by questionnaire at regularly scheduled intervals. Positive responses trigger an adverse event record, which is reviewed by the study physician for seriousness, study relatedness, and expectedness within 72 hours. Similar information reported by participants at other times (e.g., during intervention encounters) is duly noted and followed up with as needed to assure participant safety. Participants will be referred to their PCP for a medical evaluation and follow-up as needed or recommended by the study physician.

### Retention

Adherence and retention in E-LITE is fostered by: 1) selection/retention, training, and quality control of qualified staff; 2) the relationship between staff and participants, 3) willingness to accommodate participant schedules and needs, and 4) resourcefulness and persistence of the staff. A participant tracking database is used to assure that participants are contacted on a timely basis to obtain study data. The assessment staff will contact participants who miss a follow-up assessment to try re-engaging the patient in subsequent follow-ups. Ongoing monitoring of indices of participation in intervention activities helps interventionists identify participants who are having problems with adherence to the intervention protocol and thus qualify for recovery efforts. Such efforts can range from brief telephone discussion to an individualized counseling session with an interventionist in which the participant who makes a decision to discontinue intervention can review concerns regarding E-LITE, reasons for staying in E-LITE, and decisions about remaining a participant. Unless the participant declines, the interventionist will continue to contact the participant on a progressively less frequent schedule, starting monthly and decreasing to semi-annually to remind her/him of the opportunity to re-enter E-LITE and to maintain contact for possible recruitment for the final assessment at the end of E-LITE.

### Sample Size Considerations and Statistical Analysis

#### Sample Size

Given the target sample size of 240, we expect approximately 80% power for detecting a net difference of 1/2 of a standard deviation (equivalent to a medium effect size of 0.5) in BMI at 15 months between the SM intervention and usual care (and similarly, between the CM intervention and usual care) at a 2-sided α level of 0.05, assuming up to a 20% rate of loss to follow-up. The effect size is estimated based on a number of successful lifestyle weight loss intervention studies [6,18-20]. The power estimate was calculated based on a t-test using simplified assumptions. Note that the t-test calculation is conservative because it does not account for variance reduction due to adjustment for baseline variables, nor for the better than chance comparability of the experimental groups. The primary comparisons of each intervention with usual care will be conducted using two-sided procedures at the overall significant level of 0.05. Multiplicity adjustments are not necessary because the primary interest is to estimate separate treatment effects for a limited number (i.e., n = 2) of key contrasts that pertain to different interventions [53]. Therefore, p-values of each comparison will be compared to type I error α = 0.05 for significance.

### Data Analysis

The primary hypothesis compares BMI between each intervention (SM and CM) and usual care at 15 months using an intent-to-treat analysis. Linear mixed modeling,

using SAS PROC MIXED [54] will test time and group interactions to asses whether within-subject change in BMI differs by condition. This basic analytical strategy also accounts for the nature of repeated measurements, the blocked design by cohorts, and the clustering of patients within physicians. The same analysis strategy is appropriate for evaluating secondary aims where the hypotheses are identical but with different outcome measures (e.g., glycemic control, other component risk factors of the metabolic syndrome, and psychosocial well-being). The longitudinal effects of the interventions will be assessed using the same model, except that the focus is comparisons within intervention groups at different time points. Analyses will also be conducted to examine whether there is a dose-response relationship between intervention adherence and outcome, where dose is defined in terms of number of group sessions attended, number of secure messages sent, number of self-monitoring logs, average daily intakes of calories and fat, and average number of minutes of physical activity per week.

For participants with missing outcomes at the primary (15-month) or secondary (3- and 6-month) assessment point, we will conduct and report on sensitivity analyses in which different imputation methods are used (e.g., the baseline-observation-carried-forward method, the "hot deck" procedure, and an appropriate multiple imputation method [55,56].

We will use the MacArthur approach, which modified the Baron & Kenny criteria, for defining moderators and mediators in clinical research studies [57]. Let T be the treatment, M be the potential moderator or mediator, and O be the outcome. Association between T, M, and O is examined using the following models:(1)

M is a moderator of the effect of T on O if M precedes T, γ_1 _= 0, and β_3 _≠ 0. M is a mediator of the effect of T on O if T precedes M, γ_1 _≠ 0, and either β_2 _≠ 0 or β_3 _≠ 0.

### Data Management

All study data are entered into computerized data files utilizing: 1) Microsoft Access for participant tracking and intervention data entry, 2) Vovici's survey software (Dulles, VA) for self-administered questionnaires and entry of clinical measurements, 3) Food Processor, v8.7 (ESHA Research, Oregon) for nutrient analysis based on three-day food records, and 4) a custom-designed web application for seven-day physical activity recall data entry. Research assistants (blinded to treatment assignment) will review participant responses on questionnaires with participants present, and skipped or incorrectly addressed items will be brought to the participant to correct. All of the data entry systems employ automatic, real-time range, logic, and missing value checks. Data sets will be cleaned, verified and archived, and then read into SAS (Enterprise Guide 4.1; SAS Institute, Cary, NC) data sets, which also are archived. One official copy of all the study data and a master data dictionary are maintained and updated regularly by the study data analyst. All analytic and tracking database files are stored in a secure network drive with daily backups. One copy of the backups is saved on-site and one off-site. Separate archival databases are permanently maintained. The data files can be shared by authorized study personnel both on-site and in remote locations via a secure virtual private network. Multiple levels of password protection are utilized to ensure data security.

### Treatment Fidelity

Intervention quality assurance procedures ensure that project activities are standardized across the interventionists and across the cohorts and that intervention process data are collected accurately. To achieve this, standardized protocols, procedures, and educational materials are prepared, and staff are systematically trained in their use. Provider and auditor checklists are created to ensure that the intervention protocols are followed and that treatment objectives for each group session are met. Interventionists complete a Provider Checklist after each session. A random sample of 10% of group sessions will be observed and rated by a qualified individual using the Auditor Checklist. When a session is reviewed with less than 85% of treatment-specific objectives met, the auditor will inform the PI who will remediate interventionist training as needed. This process continues through all treatment waves so that interventionist drift can be swiftly and consistently corrected.

## Discussion

The U.S. health care system has evolved primarily to react to acute episodes of patient illness, and it remains largely so even though chronic disease that is largely preventable through lifestyle and behavior change has increasingly dominated patient demands and health care expenditures since the middle of the last century [58]. The enormous clinical consequences of obesity demand that our health care system resolutely take on the mission of obesity and risk factor management and integrate this task into routine clinical care. Obese patients with pre-diabetes and/or metabolic syndrome are a critical group because of their increased lifetime risk for diabetes and CVD and the potential reversibility of their condition. Yet current systems of care often fail to intervene when prevention is still possible, responding only after irreversible adverse events have occurred [59]. The standard clinical approach of delivering lifestyle modifications not only lacks in the frequency and intensity of contact to effect much change but is also missing a solid theoretical basis and behavioral self-management focus.

Efficacy trials such as the DPP [6] have shown that intensive individual lifestyle intervention results in significant weight loss and reduces the risks of type 2 diabetes and the metabolic syndrome. For intensive lifestyle interventions proven efficacious in the research setting to be successfully translated into routine clinical use, however, alternative mechanisms of delivery must be sought that are both scalable to the large, growing population of obese or overweight patients with additional risk factors and sustainable to provide ongoing patient self-management support and monitoring.

E-LITE is a randomized clinical trial aimed at rigorously evaluating the feasibility and potential effectiveness of translating two lifestyle interventions into ongoing care of overweight or obese adults with pre-diabetes and/or metabolic syndrome in a primary care practice. Studies of integrating the DPP lifestyle intervention to primary care settings are limited and few have used a randomized design [11,14,15]. In E-LITE, a DPP-based curriculum will be delivered in a self-guided DVD or in small groups, plus ongoing individual counseling via secure online messaging. Self-guided educational materials, group counseling, and secure messaging are modalities in use in contemporary health care systems. In addition to utilizing these potentially cost-effective means of delivering the intervention, the E-LITE study is designed and implemented with close attention to existing resources, skills, and barriers of the clinical setting in order to enhance its generalizability and sustainability. For example, we will recruit from an actual patient population in the clinic. We use data already available in the electronic health record to first identify an enriched pool of potentially eligible patients and then adopt a physician referral approach to promote safety and to facilitate integration of the intervention with primary care. Furthermore, we utilize well-trained non-physician clinicians (e.g., registered dietitians) who are already in the health care workforce to deliver the interventions, which will help address commonly cited barriers to primary care-based lifestyle programs in terms of physician time commitment and training. Finally, secure messaging is integrated with the electronic health record and all communications between the interventionist and the patient are viewable by the patient's PCP, which allows for integration of the intervention into ongoing care. Also, if proven successful, secure messaging would help address the need for ongoing, long-term support -- one of the greatest challenges of maintaining healthy lifestyles -- in primary care practice. With the advent of electronic health record systems and associated communication modalities, such population-based lifestyle interventions are a realistic possibility in the primary care setting.

Results from the E-LITE study will provide objective evidence on the extent of reductions in BMI and related cardiometabolic risk factors from two comprehensive, evidence-based lifestyle interventions of varying intensity that could be implemented as part of routine health care. We will also assess the potential reach, cost effectiveness, adoption, implementation, and maintenance of the interventions.

## Competing interests

The authors declare that they have no financial, research, organizational, or other interests to disclose that are relevant to the execution of this research or this publication.

## Authors' contributions

JM conceived of the study, has the overall responsibility for its design and conduct, and drafted the manuscript. ACK, SRW, RSS participated in the design of the study and revised the manuscript critically for important intellectual content. LX contributed to drafting the manuscript and to the design of the statistical analysis. All authors read and approved the final manuscript.

## Pre-publication history

The pre-publication history for this paper can be accessed here:

http://www.biomedcentral.com/1471-2296/10/71/prepub
